# Retraction Note: *In situ* structural characterization of early amyloid aggregates in Alzheimer’s disease transgenic mice and *Octodon degus*

**DOI:** 10.1038/s41598-020-76208-w

**Published:** 2020-11-10

**Authors:** Núria Benseny-Cases, Elena Álvarez-Marimon, Ester Aso, Margarita Carmona, Oxana Klementieva, Dietmar Appelhans, Isidre Ferrer, Josep Cladera

**Affiliations:** 1 ALBA Synchrotron Light Source, Carrer de la Llum 2–26, 08290 Cerdanyola del Vallès, Catalonia Spain; 2 Unitat de Biofísica i Centre d’Estudis en Biofísica, Departament de Bioquímica i de Biologia Molecular, Facultat de Medicina, Universitat Autònoma de Barcelona, 08193 Bellaterra, Catalonia Spain; 3 Biomedical Network Research Center of Neurodegenerative Diseases (CIBERNED), University of Barcelona, 08907 Hospitalet de Llobregat, Catalonia Spain; 4 Medical Microspectroscopy Research Group, Department of Experimental Medical Science, Lund University, 22180 Lund, Sweden; 5 Leibniz Institute of Polymer Research, Dresden, Hohe Strase 6, 01069 Dresden, Free State of Saxony Germany

Retraction of: *Scientifc Reports* 10.1038/s41598-020-62708-2, published online 03 April 2020

The Authors have retracted this Article.

The Authors did not have appropriate permissions for the use of the *Octodon degus* samples in this study, and therefore retract this Article.

Núria Benseny-Cases, Elena Álvarez-Marimon, Ester Aso, Margarita Carmona, Oxana Klementieva, Isidre Ferrer & Josep Cladera agree with the retraction and its wording. Dietmar Appelhans did not respond to the correspondence about the retraction.

